# Interaction with Diurnal and Circadian Regulation Results in Dynamic Metabolic and Transcriptional Changes during Cold Acclimation in Arabidopsis

**DOI:** 10.1371/journal.pone.0014101

**Published:** 2010-11-23

**Authors:** Carmen Espinoza, Thomas Degenkolbe, Camila Caldana, Ellen Zuther, Andrea Leisse, Lothar Willmitzer, Dirk K. Hincha, Matthew A. Hannah

**Affiliations:** Max-Planck-Institute of Molecular Plant Physiology, Potsdam, Germany; USDA-ARS, United States of America

## Abstract

In plants, there is a large overlap between cold and circadian regulated genes and in Arabidopsis, we have shown that cold (4°C) affects the expression of clock oscillator genes. However, a broader insight into the significance of diurnal and/or circadian regulation of cold responses, particularly for metabolic pathways, and their physiological relevance is lacking. Here, we performed an integrated analysis of transcripts and primary metabolites using microarrays and gas chromatography-mass spectrometry. As expected, expression of diurnally regulated genes was massively affected during cold acclimation. Our data indicate that disruption of clock function at the transcriptional level extends to metabolic regulation. About 80% of metabolites that showed diurnal cycles maintained these during cold treatment. In particular, maltose content showed a massive night-specific increase in the cold. However, under free-running conditions, maltose was the only metabolite that maintained any oscillations in the cold. Furthermore, although starch accumulates during cold acclimation we show it is still degraded at night, indicating significance beyond the previously demonstrated role of maltose and starch breakdown in the initial phase of cold acclimation. Levels of some conventional cold induced metabolites, such as γ-aminobutyric acid, galactinol, raffinose and putrescine, exhibited diurnal and circadian oscillations and transcripts encoding their biosynthetic enzymes often also cycled and preceded their cold-induction, in agreement with transcriptional regulation. However, the accumulation of other cold-responsive metabolites, for instance homoserine, methionine and maltose, did not have consistent transcriptional regulation, implying that metabolic reconfiguration involves complex transcriptional and post-transcriptional mechanisms. These data demonstrate the importance of understanding cold acclimation in the correct day-night context, and are further supported by our demonstration of impaired cold acclimation in a circadian mutant.

## Introduction

Cold negatively affects plant growth and development. Many temperate plants, including *Arabidopsis thaliana*, increase their freezing tolerance after exposure to low, but non-freezing temperatures, in a process termed cold acclimation. During cold acclimation, plants display a wide reprogramming of gene expression [1], [2], [3], [4], [5], [6], [7]. At the transcriptional level, the *C-repeat binding factors* (CBFs)-*1*, -*2* and *3* are the best characterized regulators of cold acclimation. *CBFs* are rapidly induced in cold, coordinating the expression of the “*CBF* regulon” which has a large effect on freezing tolerance [8], [9]. However, transcript profiling data suggest the induction of multiple transcriptional pathways [3], [10]. Several studies have characterized the massive metabolic changes induced by cold [4], [5], [7], [11], [12], [13], [14], [15]. These studies showed extensive modifications supporting cellular changes, increased antioxidant production and the accumulation of compatible solutes such as raffinose and proline. An upregulation of the urea and citric acid cycles, as well as accumulation of trehalose and putrescine have been suggested to have a role in freezing tolerance [12]. *CBFs* have a prominent role in this reconfiguration, with around 80% overlap between metabolic responses of constitutive overexpression of *CBF3* and cold acclimation [11]. Metabolic reconfiguration during cold is also dependent on stress duration [14] and developmental stage [14], [15].

Changes in the environment are coordinated with internal processes through the circadian clock [16], [17] which is composed of a complex network of interlocking feedback loops [18]. Circadian rhythms generated by the clock persist in constant conditions with an approximately 24 h period, and can be entrained by environmental cues such as light-dark and temperature cycles. Circadian clocks are able to maintain stable rhythms over a broad range of physiological temperatures, a process termed “temperature compensation” [17]. Estimates of the number of circadian-regulated genes in Arabidopsis range from hundreds [19] to essentially all expressed genes [20]. Among the circadian-regulated genes are the *CBF*s and several cold-regulated (*COR*) genes [1], [19]. A consequence of circadian regulation is that the level of stimulus-dependent transcript accumulation can be strongly influenced by the time of day at which the stimulus is applied, a phenomenon known as “gating”. Gating of cold-induction has been shown for *CBF1*, *CBF2* and *CBF3* expression, as well as for the cold-responsive transcription factors *RAV1* and *ZAT12* [21], and for the expression of *COR78* [22]. Recently, it was shown that cold gating affects the strength of expression changes of more than 50 transcription factors and additionally that time of day makes a substantial contribution to inter-experiment variation on the identity of cold responsive genes [1]. The reason for this time-of-day dependence is that under light-dark cycles (L/D) cold dampens the cycles of clock oscillator components and disrupts those of some circadian output genes, while under circadian conditions (L/L; continuous light) oscillator components stop cycling under cold [1]. Similarly, circadian clock function is disrupted in chestnut during winter dormancy [23], [24]. Furthermore, time-of-day has also emerged as a factor that can more generally affect stress responses, as demonstrated for drought [25], or by recent work on the effect of circadian clock function on metabolism during abiotic stress [5], [26], [27].

In spite of these observations, the consequences of such interactions for the complex molecular and physiological changes that accompany cold acclimation remain unclear. Time dependence of transcriptional [6] and metabolic [12], [14] changes during cold acclimation has been widely recognised but only in a simplified linear sense i.e. short-, medium-, and long-term effects. However, the dramatic influence of time in the diurnal, i.e. cyclical, context on identification of cold responsive genes has only rarely been considered [1].

In the present study, we performed transcript profiling together with the analysis of primary metabolites to better understand the response to cold during light-dark cycles and in continuous light time series. In this integrated study we survey for changes in metabolite levels and investigate whether these are consistent with changes in transcript abundance as a factor in configuring plant metabolism in response to cold. We demonstrate that diurnal regulation results in dynamic molecular changes during cold acclimation; and this metabolic reconfiguration involves transcriptional regulation, mostly associated with “conventional” cold-stress metabolites, such as raffinose, proline and γ-aminobutyric acid (GABA). For other molecules, more complex regulatory mechanisms are involved.

## Results

### Experimental design

**Figure 1:**
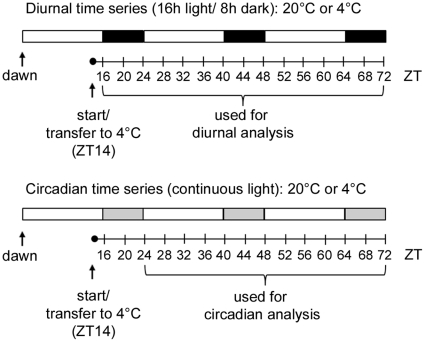
Experimental design of diurnal and circadian time series. Whole rosettes were harvested at the indicated ZT (zeitgeber time, in hours) and used for transcript and metabolite profiling. Temperature and light conditions are indicated. White, black and grey bars indicate the corresponding day, night and subjective night periods.

Metabolite and transcript profiling were performed on soil-grown plants (standard compost mix – see Methods), sampled from four different conditions. Time series were harvested under light-dark cycles (L/D; 16/8 h) and continuous light (L/L, 24 h) at control temperature (20°C) and after transfer to 4°C for cold acclimation. Long-day conditions were used for consistency with previous studies of metabolic changes associated with cold acclimation [4], [5], [7], [14] and previous work on interactions between cold and the circadian clock [1], [23], [24]. These studies, including our own work, indicate that under these experimental conditions Arabidopsis cold acclimates, metabolic changes occur and the expression of clock components is disrupted. The experiment was started 2 h before (subjective) dusk and leaves were sampled at time 0 (ZT14) and after 2 h (to coincide with (subjective) light-dark transitions) and then every 4 h until 58 h (ZT72) (Figure 1). Previous analyses already demonstrated that gene expression becomes arrhythmic under L/L in the cold [1]. Therefore, the L/L 4°C time series was not analyzed by transcript profiling, while all four conditions were analyzed by metabolite profiling, yielding a total of seven time course datasets. To identify transcripts and metabolites under circadian control we used the subset of our L/L 20°C condition from 10 h to 58 h (corresponds to Zeitgeber Time, ZT24 – ZT72), in agreement with previous studies [19]. It should be noted that in the context of circadian regulation our 4°C conditions include the adjustment phase, where cycles observed from 0 h to 24 h could still be attributed to free-running cycles established before transfer. Transcript profiling was performed using Affymetrix ATH1 arrays and metabolite profiling of polar metabolites by GC-MS. Following normalization and filtering (see Methods) our final dataset included transcripts corresponding to 14874 genes and 50 metabolites. In addition, starch content was determined by an enzymatic assay.

### Temperature and light-dark cycles cause extensive changes in transcripts and metabolites

**Figure 2:**
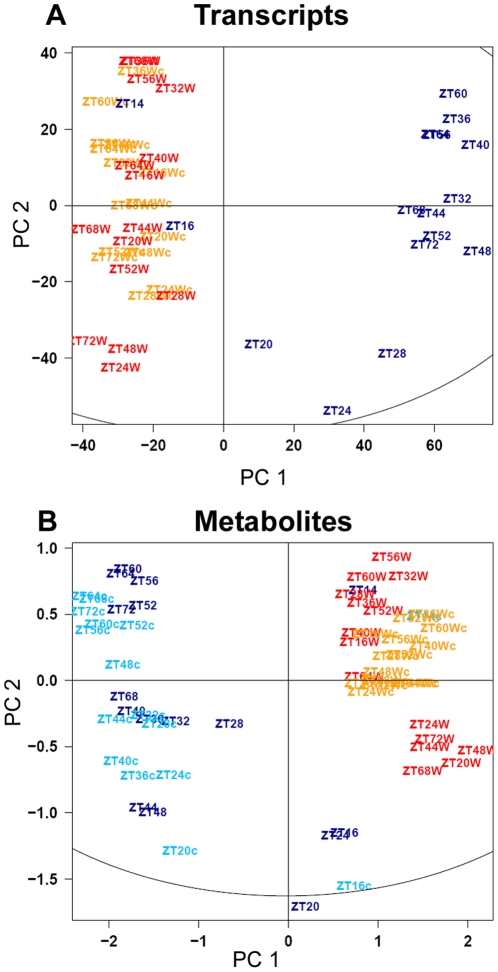
Contribution of low temperature and diurnal regulation to the variation in transcripts and metabolites in diurnal and circadian time courses. PCA (Principal Component Analysis) was applied to transcript (A) and metabolite (B) profiling datasets from diurnal and circadian time courses. The color indicates the different light and temperature conditions of the studied time courses: light/dark cycles either at 20°C (red) or 4°C (dark blue) and continuous light at 20°C (orange) or 4°C (light blue). Circadian time courses are denoted by c in lowercase. Sampling time is indicated by ZT (zeitgeber, in hours). PC1 and PC2 correspond to principal component 1 and 2, respectively. Each point in (A) represents a single transcript profile, whilst in (B) it represents the mean metabolite profile of five biological replicates.

Principal Component Analysis (PCA) was used to determine the main contributions to variation in the data. Figure 2 shows that temperature was responsible for the primary difference in both transcript and metabolite datasets. Principal Component 1 (PC1) separates samples of cold treated plants from those at 20°C, and explains 38% and 67% of the variation for the transcript and metabolite data, respectively. Time zero samples (ZT14) clustered together with samples at 20°C, and subsequent time points from the cold series showed progressively larger separation from time 0, as expected from previous publications [5], [12], [14]. Interestingly, in L/L separation of samples based on metabolite changes in the cold progressed more rapidly than in L/D, with earlier time points segregating more strongly from the control samples. Diurnal variation was also clearly visible and underlies PC2, which explains 13% and 12% of the variation for the transcript and metabolite data, respectively. In case of transcripts, diurnal patterns are apparent in Figure 2 where samples from time points that differ by 24 h mostly group together in the L/D 20°C time course. In addition, L/D and L/L samples at 20°C are also separated by PCA as L/L samples are more tightly grouped than the L/D samples, highlighting the greater contribution of diurnal regulation to sample variation. For the metabolites, where both L/L and L/D were additionally studied in the cold, the contribution of diurnal regulation was less pronounced at 4°C than 20°C, consistent with a disruption of the clock by cold [1].

### Effect of cold on the cycling of transcripts and metabolites

**Figure 3:**
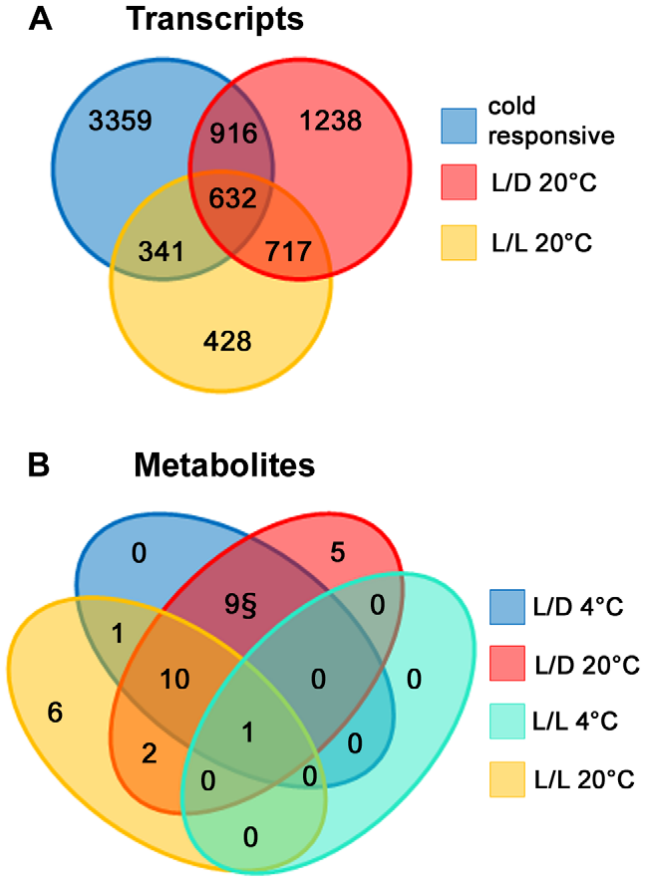
Effect of cold on cyclic gene expression and metabolite content. (A) Venn diagram showing the number and overlaps between of transcripts classified into each of the following three groups: 1) diurnally regulated at 20°C, 2) circadian regulated at 20°C or 3) cold-responsive. Circadian and diurnal regulated transcripts were identified using the HAYSTACK tool or by autocorrelation, respectively. Cold responsive genes were identified using limma with a sliding window of three sequential time points (FDR<0.05) (see Methods for details). (B) Venn diagram showing the number and overlaps between metabolites classified into each of the following four groups: 1) diurnally regulated at 20°C, 2) circadian regulated at 20°C, 3) diurnally regulated at 4°C, 4) circadian regulated at 4°C. Circadian and diurnal regulated transcripts were identified using the HAYSTACK tool or by autocorrelation, respectively (see Methods for details).

Previous studies of diurnal or circadian regulation of gene expression [19], [20], [28], [29] mostly used 12 h photoperiod, and in some cases, plants grown on agar plates (with sugar). Furthermore, there have been very few metabolite profiling studies on diurnal or circadian regulation [30]. As mentioned above, for consistency to previous work on cold acclimation and the effect of cold on the circadian clock, we used 16 h photoperiod and soil grown plants. Therefore, we used our L/D 20°C and L/L 20°C control datasets to identify rhythmic transcripts and metabolites and the phase of their maximal accumulation (see Methods). This indicated that of 14874 detected transcripts, 3507 (24%) cycled in L/D at 20°C while 2119 (14%) showed free-running cycles in L/L 20°C consistent with circadian regulation (Figure 3A and Table S2). Of the genes that were classified as having circadian regulation in L/L at 20°C, 64% also show diurnal oscillations at this temperature (overlap  = 1349 genes, Figure 3A). It should be noted that complete overlap is not expected due to unavoidable false negatives in both gene lists resulting from the use of defined correlation and amplitude cut-offs. Comparison of the L/D 4°C to the L/D 20°C dataset revealed a total of 5251 genes were cold responsive (FDR<0.05), and this group of genes from the L/D 4°C dataset was subjected to k-means clustering in order to visually determine the effect of cold over the light-dark cycles. Although there were more than 1500 genes that were both cold responsive and cycled under L/D 20°C (Figure 3A), the clustering only identified a subgroup of 376 genes that showed clear diurnal oscillations at 4°C (Table S1) indicating that only a small proportion of the genes cycling under L/D at 20°C maintained clear diurnal oscillations at 4°C.

Twenty-four of the identified metabolites (47%) exhibited significant diurnal oscillations at 20°C, while 19 metabolites (37%) showed significant circadian cycles (Figure 3B and Table 1), showing that 63% of the metabolites showing circadian cycles at 20°C also cycle in L/D at 20°C (overlap  = 12 metabolites). During cold acclimation, 80% of these diurnally regulated metabolites kept their oscillations, although not all at the same phase. Interestingly, maltose was the only metabolite that maintained any circadian oscillation in the cold, although only during the first day following transfer to 4°C. Thus, there was a significant effect of low temperature on the circadian regulation of metabolite content, similar to the massive effect of cold observed at the transcriptional level.

**Table 1:** Summary of the changes in primary metabolism during cold acclimation in diurnal and circadian time courses.

	Diurnal (L/D)		Circadian (L/L)	
Metabolites	20°C	4°C	20°C	4°C
	(phase^A^/correlation^B^)		(phase^A^/correlation^B^)	(pattern)
Lysine	0/0.789	Down, no cycles	11/0.749	Down, no cycles
Fructose	4/0.872	Up, cycles^C^	22/0.771	Up, no cycles
Sorbose	4/0.859	Up, cycles	21/0.722	Up, no cycles
Glucose	4/0.776	Up, cycles^C^	X	Up, no cycles
Ornithine	4/0.726	Up, cycles^C^	3/0.713	Up, no cycles
Raffinose	4/0.735	Up, no cycles	x	Up, no cycles
Serine	8/0.852	No cycles	x	Up, no cycles
Beta-alanine	8/0.826	Up, cycles^E^	12/0.766	Up, no cycles
Homoserine	12/0.974	Up, cycles	x	Up, no cycles
Methionine	12/0.963	Up, cycles	x	Up, no cycles
Arginine	12/0.912	Up, cycles	x	Up, no cycles
**Isoleucine**	**12/0.869**	**Cycles** F	**11/0.797**	**No changes**
**Leucine**	**12/visual** D	**No change**	**11/0.730**	**Up, no cycles**
**Valine**	**12/visual** D	**Up, cycles**	**13/0.726**	**Up, no cycles**
Glycine	12/0.804	Up, cycles	23/0.720	Up, no cycles
GABA	14/visual^D^	Up, no cycles	x	Up, no cycles
Starch	16/visual^D^	Up, cycles	x	Up, no cycles
**Alanine**	**16/0.951**	**Up, cycles**	**15/0.717**	**Up, no cycles**
Pyruvic acid	16/0.945	No changes	x	Down, no cycles
Phenylalanine	16/0.926	Up, cycles	19/0.782	Up, no cycles
Tyrosine	16/0.901	Up, cycles	x	Up, no cycles
2-OG	16/0.894	No cycles	x	No changes
**Glutamine**	**16/0.723**	**Up, cycles**	**18/0.717**	**Up, no cycles**
**Maltose**	**20/0.997**	**Up, cycles** G	**21/0.766**	**Up, cycles**
**OAS**	**20/0.830**	**Up, cycles**	**23/0.801**	**Up, no cycles**
Trehalose	20/0.766	Up, cycles	x	Up, no cycles
Putrescine	x	Up, no cycles	23/0.711	Up, no cycles
**Fucose**	**x**	**Up, no cycles**	**3/0.701**	**Up, no cycles**
Nicotinic acid	x	Down, no cycles	11/0.773	Down, no cycles
Galactinol	x	Up, no cycles	x	Up, no cycles
Proline	x	Up, no cycles	1/0.716	Up, no cycles
Asparagine	x	Up, no cycles	12/0.705	Up, no cycles

The table includes metabolites with diurnal and circadian oscillations, as well as conventional cold induced metabolites. Metabolites with circadian cycles confirmed by an independent experiment are highlighted in bold.

(A) Phase related to best model fitting experimental data.

(B) Correlation indicates Pearson's correlation coefficient derived by comparison of the experimental data series and the best-fitting model using the HAYSTACK algorithm or the average autocorrelation (see Methods).

(C) Diurnal oscillations are shifted in cold.

(D) Visual indicates that a metabolite cycles as determined by manual inspection rather than the statistical cutoff.

(E) Cycles with inverted phase.

(F) Cycles with lower amplitude.

(G) Cycles with higher amplitude.

### Diurnal and circadian regulation of primary metabolism

**Figure 4:**
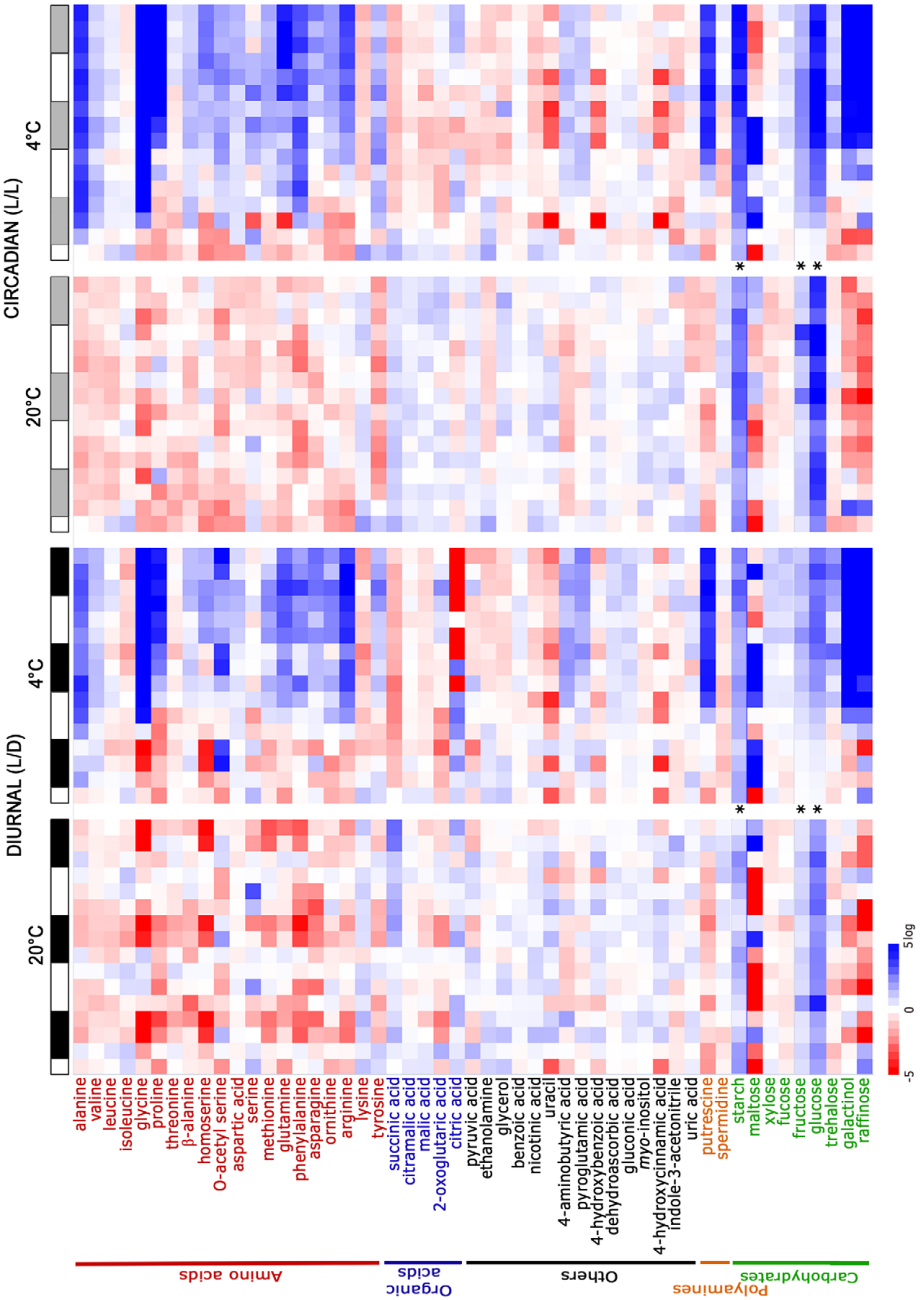
Global overview of the dynamic changes in primary metabolism during cold acclimation in diurnal and circadian time courses. Heatmaps showing the changes occurring across time in the pool sizes of different metabolites during the four performed time courses. From left to right, these represent 16 h/8 h light/dark at 20°C, 16 h/8 h light/dark at 4°C, continuous light at 20°C and continuous light at 4°C. Along the top axis white bars indicate light, black bars indicate dark and grey bars indicate the subjective dark periods (i.e. corresponding to the entrainment conditions). Metabolite levels are the mean log2 values from five biological replicates depicted in a false color scale where red indicates low and blue indicates high values in the range of −5 to +5 (log2). *Due to the high levels of glucose, fructose and starch content at low temperature in comparison to other metabolites, the false color scale ranges from −15 to +15 (log2). As not all cyclical regulation is visible in this figure (especially where a metabolite is strongly cold responsive), it should be interpreted in conjunction with the detailed classification of circadian and diurnal regulation of the different metabolites shown in Table 1.

Our metabolite analysis focuses on primary metabolism, particularly sugars, amino acids and organic acids as these are well-represented in GC-MS data sets and have thus been extensively discussed in the literature. A detailed classification of metabolite pool sizes based on their behaviour in L/D and L/L under the temperature conditions studied was performed (Table 1). In L/D conditions at 20°C, the cycles in pool sizes of those metabolites identified computationally as having diurnal cycles at 20°C are clearly observable in the heatmap representation of all conditions (Figure 4), for example glycine, alanine, homoserine and methionine. When plants were transferred to cold the diurnal cyclic pattern was affected differently for different metabolites. Maltose, phenylalanine, tyrosine and methionine accumulated in the cold and the cyclic changes in content were maintained. In contrast, fructose, glucose and ornithine also accumulated at 4°C, but the phases of their cycles were shifted. Similar diversity was observed for metabolites that decreased in the cold. For instance, some preserved their diurnal oscillations, such as isoleucine, while others stopped cycling, like lysine. On the other hand, if oscillations of metabolite accumulation are due to circadian regulation, cycles should persist in L/L. Examples of this response were observed for maltose, O-acetyl serine (OAS), valine and alanine at 20°C. At low temperature their content either increased (phenylalanine), or decreased (lysine), but only maltose preserved any oscillation in L/L at 4°C. Some metabolites, such as putrescine and fucose, did not diurnally oscillate, but cycled in L/L at 20°C, thus showing circadian regulation. Their pool sizes also strongly increased in response to cold. Finally, some conventional cold-responsive metabolites, such as raffinose showed diurnal cycles in plants at 20°C, while galactinol and proline showed no evidence of cycling.

### Diurnal and circadian time series show contrasting metabolic correlations

The previous analyses focused on diurnal and circadian cycles in metabolite content at the population level (i.e. the median of five plants). Coordinated regulation of metabolite pool sizes in individual plants was not considered in this approach. Therefore, we also analyzed changes in metabolite content at the individual plant level by identifying metabolite pairs that change concordantly over time in all plants, without the prerequisite that they are at the same level or have the same phase of cycling in all plants. These correlations were used to construct undirected metabolic networks (Figure S1) where nodes represent metabolites and the edges (i.e. connecting lines) represent highly significant pairwise Spearman correlations. Selected global topological network parameters were calculated and compared between networks (Table 2). All networks had a similar diameter; however, in L/D the networks were consistently more connected than in L/L, indicating that in L/D metabolic changes were more coordinated (i.e. correlated) at an individual plant level. Also, the loss of cycling found for many metabolites in L/L reflects an overall lower coordination of metabolism. The heterogeneity of the networks (tendency to contain highly connected hubs) was higher in L/D compared to L/L, indicating that L/D conditions lead to a more integrated regulation of metabolism than continuous light. This topological difference was maintained at 4°C. Still, the response to cold clearly influenced the network parameters. The number of connections (i.e. edges) was higher at 4°C in L/L and L/D, respectively, resulting in a higher density of these networks, suggesting that the massive reprogramming of metabolism occurring at 4°C was highly coordinated. This is further indicated by the higher centrality of these networks (Table 2), indicating more highly connected metabolites.

**Table 2:** Selected network parameters of metabolite correlation networks.

	Diurnal (L/D)		Circadian (L/L)	
Network parameter	20°C	4°C	20°C	4°C
Number of nodes (metabolites)	57	59	43	54
Number of edges (significant metabolite-metabolite correlations)	121	205	66	179
Network diameter (longest distance between nodes in network)	8	7	8	7
Connected Pairs/number of shortest paths	1434	2462	886	2454
Average number of neighbors (average number of significant correlations per metabolite)	4.25	6.95	3.07	6.63
Characteristic path length (expected distance of two connected nodes)	3.11	2.69	3.59	2.79
Network density (average number of neighbors normalized to the number of nodes)	0.08	0.12	0.07	0.13
Centralization (tendency of the network to contain central nodes)	0.16	0.25	0.12	0.24
Network heterogeneity (tendency of the network to contain highly connected nodes)	0.80	0.89	0.63	0.72
Clustering coefficient (tendency of the nodes to cluster together)	0.33	0.45	0.47	0.46

The most important topological parameters for the metabolic networks corresponding to all conditions studied. In the networks nodes (representing metabolites) were connected with lines (edges) if their pairwise Spearman correlation was significant (Bonferroni corrected p-value <0.001) under the respective experimental condition. Topological parameters were calculated with the Cytoscape plugin Network Analyzer.

**Figure 5:**
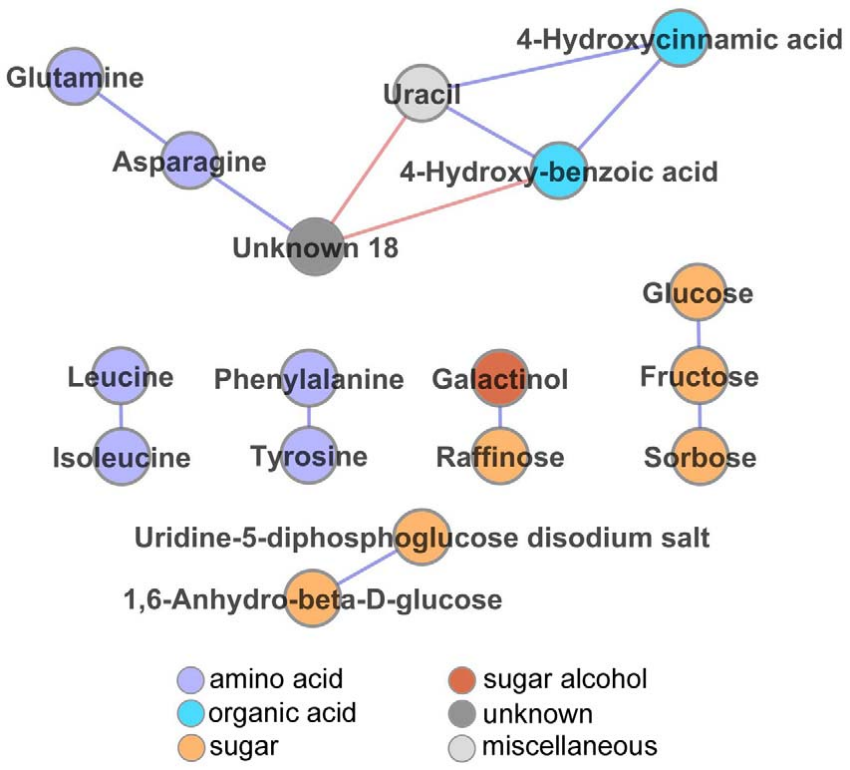
Core network of metabolite correlations observed in diurnal and circadian conditions both at 20°C and 4°C. In the depicted network, nodes (spots) represent metabolites and edges (lines) indicate highly significant positive (blue) and negative (red) pairwise correlations between metabolites. The core network represents thirteen correlations between seventeen metabolites which were stably in diurnal and circadian conditions both at 20°C and 4°C (i.e. in all four studied time series). Node color codes indicate compound classes as described in the figure. The significance threshold of the Spearman correlations was set at <0.001 for the Bonferroni corrected p-values (see Methods).

In addition, the core network, representing the metabolite-metabolite correlations that were stably present under all experimental conditions, revealed biologically relevant modules (Figure 5). Thirteen edges connecting 17 metabolites were present under all experimental conditions, such as glutamine – asparagine, raffinose – galactinol, phenylalanine – tyrosine, leucine – isoleucine, glucose – fructose and 4-hydroxycinnamic acid – 4-hydroxybenzoic acid. In the 20°C L/L network an amino acid-dominated module was present consisting of branched-chain (isoleucine, leucine and valine) and aromatic (phenylalanine and tyrosine) amino acids, beta-alanine and lysine. In L/D they were additionally connected to other amino acids as well as organic acids such as 2-oxoglutaric acid, pyruvic acid and glycolic acid. Maltose was positively correlated to amino acids in L/L, whereas it was negatively correlated in L/D. Under both light conditions the known stress responsive metabolites raffinose, putrescine, proline, galactinol, trehalose and GABA were more highly connected at 4°C than at 20°C, indicating coordinated stress responses of metabolism.

### Diurnal, circadian and cold-regulation of characteristic cold-induced metabolic pathways

Among the well-characterised metabolic responses to cold is the accumulation of compatible solutes and of transcripts encoding enzymes from their biosynthetic pathways [12].

**Figure 6:**
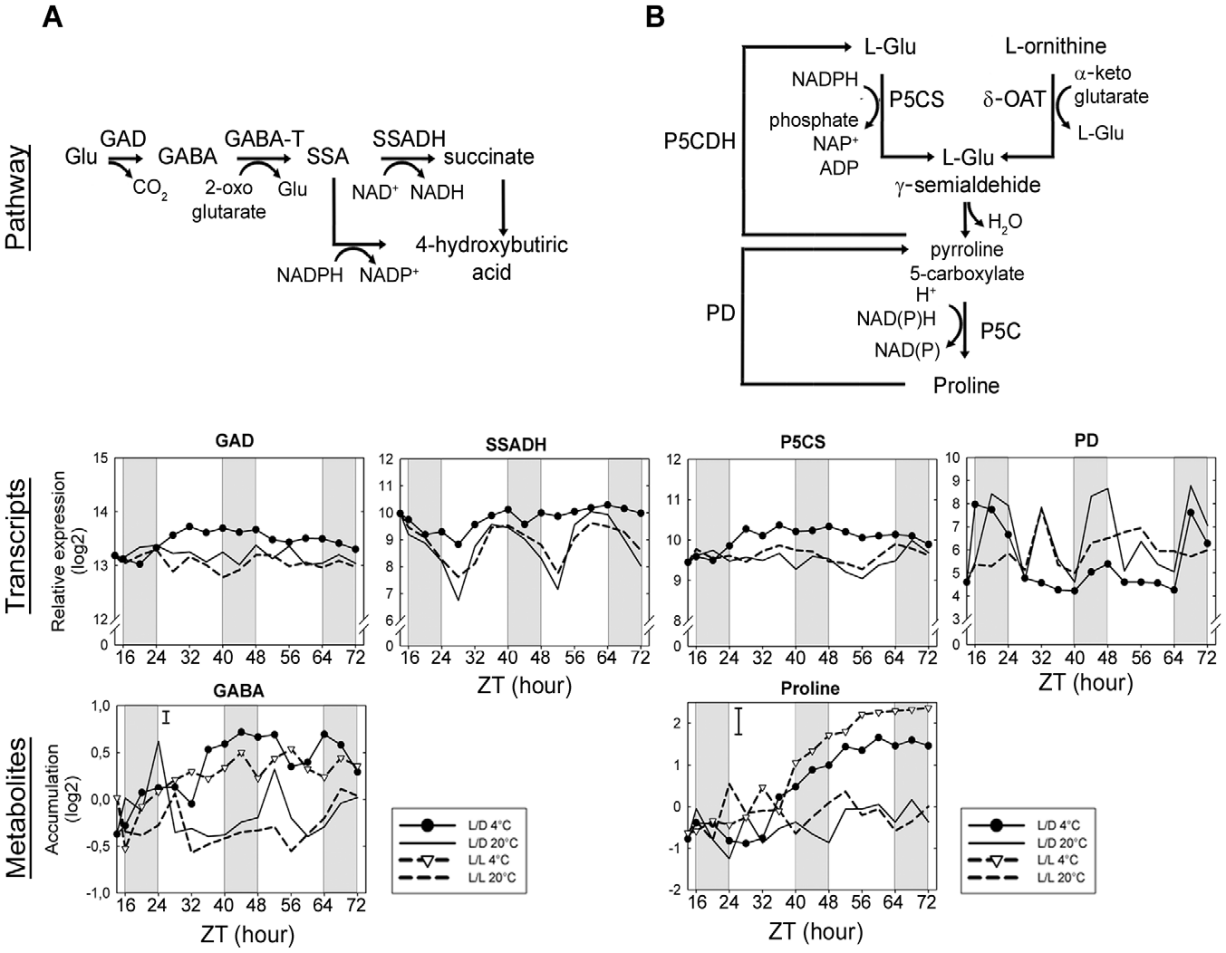
Coordinated transcriptional regulation of conventional cold induced metabolites. Summary of the metabolic pathway, transcript and metabolite profiles of GABA (A) and proline (B). For transcripts, relative expression (log2) from a pool of five biological replicates is indicated. Metabolite content (log2) corresponds to the normalized peak apex intensities from five biological replicates with bars indicating the largest standard deviation. GAD, glutamate decarboxylase (*At1g65960*); GABA-T, GABA transaminase; SSADH, succinic semialdehyde dehydrogenase (*At1g79440*); SSA, succinate semialdehyde; Glu, glutamate; GABA, 4-aminobutyrate; P5CS, Δ1-pyrroline-5-carboxylate synthase (*At2g39800*); δ-OAT, ornithine-δ-aminotransferase; P5C, pyrroline-5-carboxylate reductase; PD, proline dehydrogenase (*At3g30775*); P5CDH, 1-pyrroline-5-carboxylate dehydrogenase.

GABA levels showed an apparent diurnal regulation, with a phase at or closely following dawn and oscillations with delayed phase and reduced amplitude in continuous light at 20°C (Figure 6A). At low temperature, an increase in the amount of the transcript encoding glutamate decarboxylase (*GAD*), responsible for GABA biosynthesis, preceded the increase in GABA content which peaks around 30 h (ZT44). Subsequent decreases in GABA levels also corresponded to a decrease in *GAD*. Expression of the genes encoding GABA transaminase (*GABA-T*) (Table S2) and succinic semialdehyde dehydrogenase (*SSADH*) involved in GABA catabolism exhibited diurnal and circadian oscillations at 20°C, but diurnal oscillations of *SSADH* were stopped in the cold, keeping the expression at its maximum level (Figure 6A).

In nonacclimated plants, proline showed circadian oscillations but no diurnal oscillations were observed (Figure 6B). The increase in proline content during cold acclimation was preceded by an induction of the transcript for its biosynthetic enzyme Δ^1^-pyrroline-5-carboxylate synthase (*P5CS*), consistent with transcriptional regulation. A potential role for reduced degradation was also observed, with a down regulation of proline decarboxylase (*PD*) in the early phases of cold treatment. *PD* showed an interesting diurnal regulation with two daily peaks – one in the middle of the day and another in the middle of the night, with diurnal rather than circadian regulation apparently contributing to the first peak.

Other cold-stress metabolites also showed coordination between transcripts and metabolites, such as galactinol, raffinose and putrescine (Figure S2). In summary, increases in the transcripts encoding metabolic enzymes of conventional cold-stress metabolites preceded the accumulation of the corresponding metabolites, thus showing a coordinated transcriptional regulation during cold acclimation. Moreover, those transcripts were often diurnal and/or circadian regulated.

### Dynamic responses of transcripts and metabolites at low temperature

Contrary to the solutes described above, less is known regarding the regulation of other metabolites during cold acclimation. We therefore determined the regulation of different cold-responsive metabolites and investigated potential correspondence between transcripts and metabolites.

**Figure 7:**
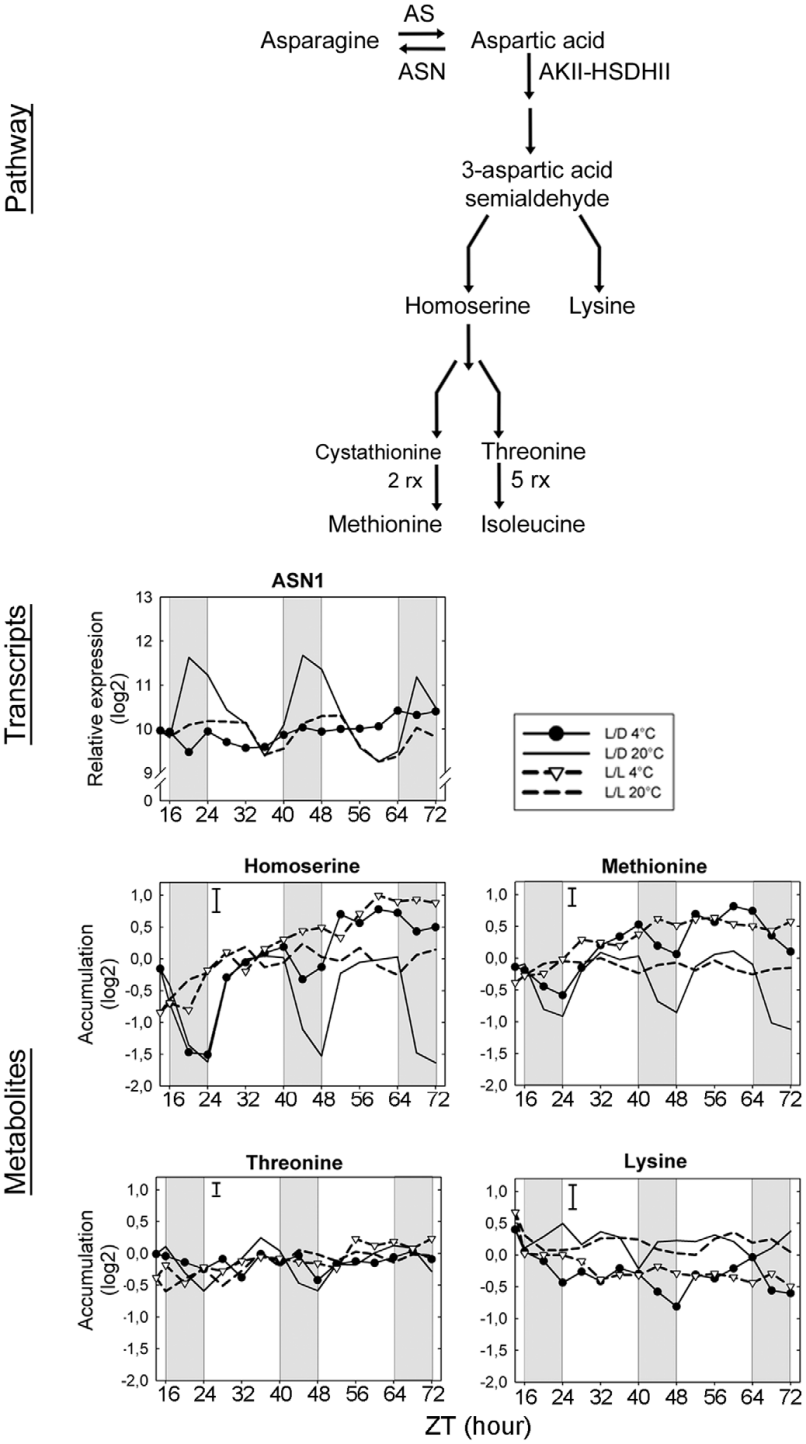
Integration of gene expression and metabolite accumulation for the aspartic acid biosynthetic pathway. For transcripts, relative expression (log2) from a pool of five biological replicates is indicated. Metabolite content (log2) corresponds to the normalized peak apex intensities from five biological replicates with bars indicating the largest standard deviation. AS, asparaginase; ASN, asparagine synthase (ASN1: *At3g47340*); AKII-HSDHII, aspartate-kinase-homoserine-dehydrogenase.

The aspartate pathway has two branches, one leading to the synthesis of lysine, and the other to isoleucine, threonine and methionine through the common precursor homoserine. In nonacclimated plants, homoserine, methionine and threonine levels showed parallel diurnal oscillations peaking around midday (Figure 7), but no circadian regulation. At 4°C, this coordination was not observed. Instead, homoserine and methionine levels increased, but threonine content remained constant. On the other hand, circadian in addition to diurnal oscillations were observed for lysine levels in plants at 20°C (Table 1) and a decrease during cold acclimation was evident. Aspartic acid is the common precursor of both branches of the pathway and can also be converted to asparagine by asparagine synthase (*ASN*) [31]. Transcripts of *ASN1* showed diurnal and circadian oscillations at 20°C. At 4°C, diurnal cycles of *ASN1* expression were dampened to the lowest diurnal or circadian level. However, the contribution of this regulation to the availability of aspartic acid remains to be established.

**Figure 8:**
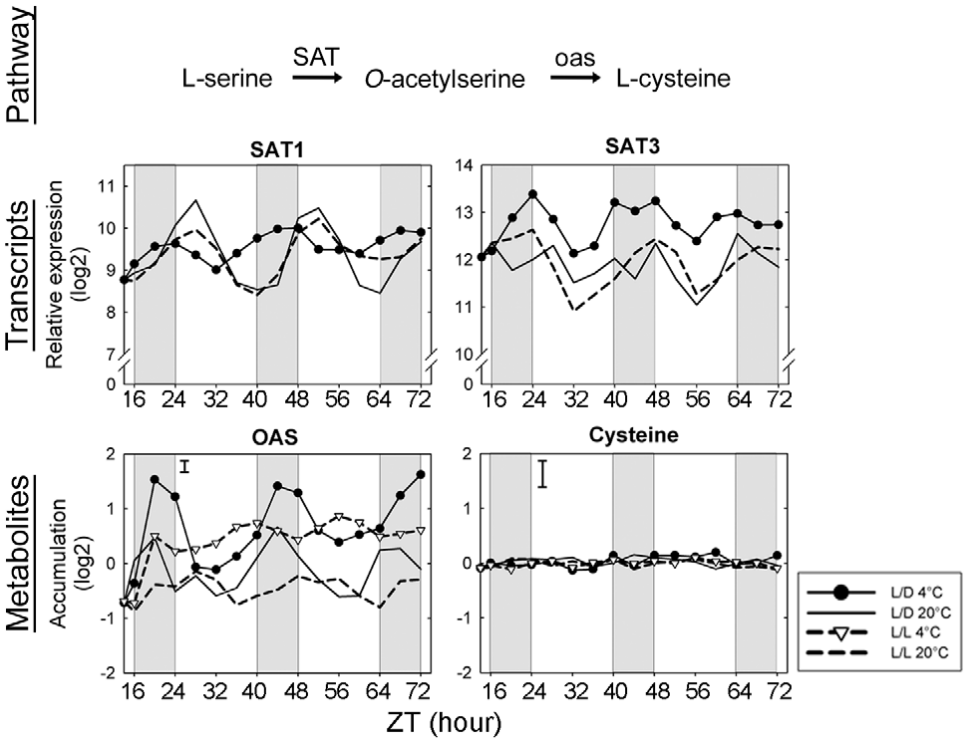
Integration of gene expression and metabolite accumulation for OAS. For transcripts, relative expression (log2) from a pool of five biological replicates is indicated. Metabolite content (log2) corresponds to the normalized peak apex intensities from five biological replicates with bars indicating the largest standard deviation. SAT, serine O-acetyltransferase (SAT1: *At1g55920*; SAT3: *At3g13110*); oas, cysteine synthase; OAS, *O*-acetylserine.

OAS is a direct precursor of cysteine. At 20°C, OAS pool sizes exhibited diurnal and circadian oscillations, peaking during the dark period (Figure 8). During cold treatment, levels of OAS strongly increased within a few hours and diurnal oscillations were observed. OAS is synthesized from serine by O-acetyltransferase (*SAT*). In plants at 20°C, *SAT1* and *SAT3* showed diurnal and circadian oscillations in expression with different phases. Interestingly, the peak expression of *SAT3* occurred at the same phase as the maximum OAS levels. During cold acclimation, *SAT3* expression increased, and *SAT1* accumulation changed its phase. No changes in cysteine levels were observed (Figure 8) and several genes coding for cysteine synthase showed decreasing levels during cold acclimation (*At2g43750*, *At3g59760*, *At4g14880* and *At5g28020*) (Table S2 *).*

The branched-chain amino acids are highly coupled at 20°C, while they are clearly separated at 4°C. Some transcriptional changes may contribute to increased valine content but are too slow to be the initial trigger for it (Figure S3).

**Figure 9:**
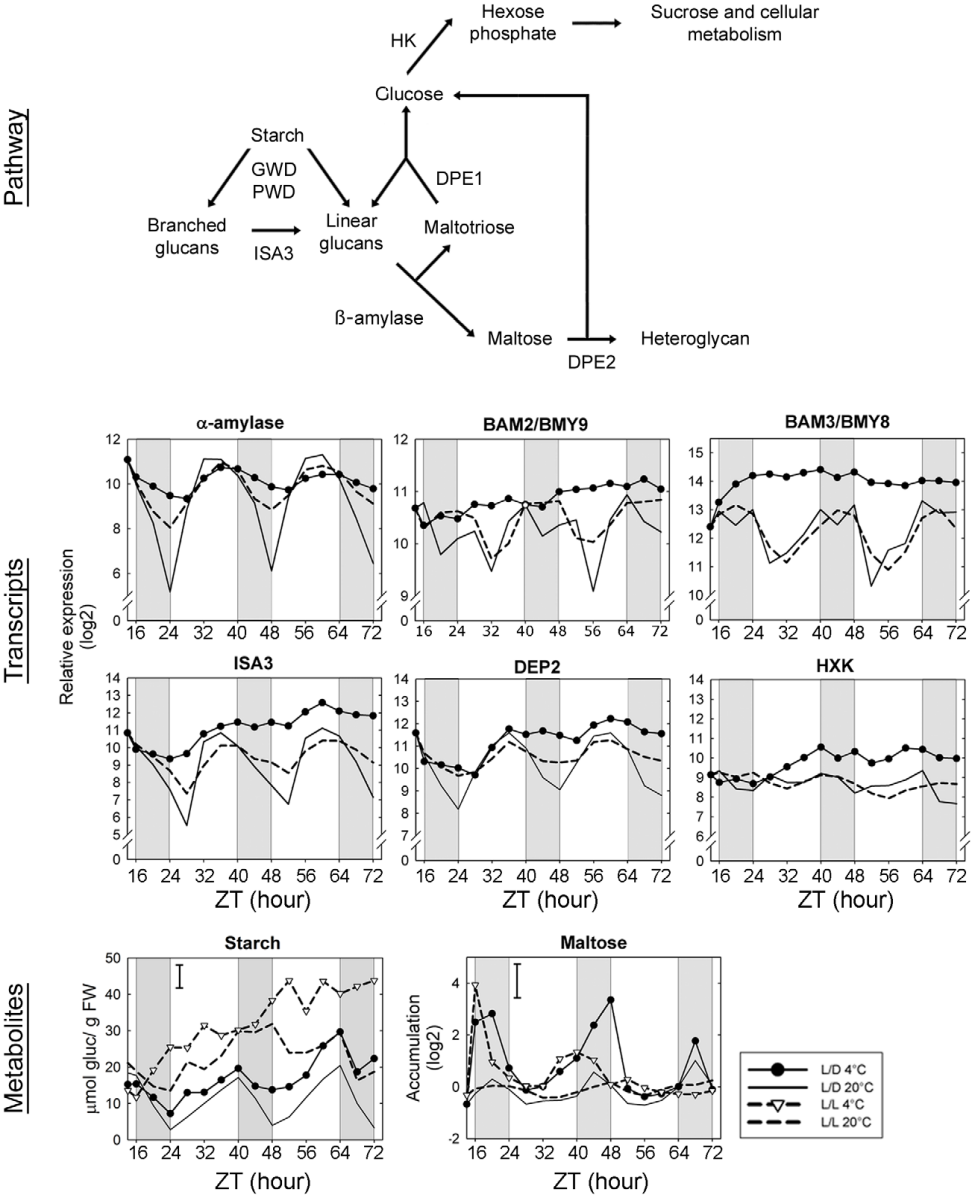
Integration of gene expression and metabolite accumulation for starch degradation pathway. For transcripts, relative expression (log2) from a pool of five biological replicates is indicated. Metabolite content (log2) corresponds to the normalized peak apex intensities from five biological replicates. GWD, glucan water dikinase; PWD, phosphoglucan, water dikinase; ISA3, isoamylase3 (*At4g09020*); DPE1, disproportionating enzyme; DPE2, cytosolic transglucosidase (*At2g40840*). HK, hexokinase (*At2g19860*); α-amylase (*At1g69830*); BAM2/BMY9, β-amylase (*At4g00490*); BAM3/BMY8, β-amylase (*At4g17090*).

Consistent with previous reports, starch content showed diurnal cycles at 20°C and our data show these were maintained at 4°C, albeit with a raising baseline. However, cycles were not maintained in L/L at either temperature (Figure 9). Maltose levels showed diurnal oscillations at 20°C with the highest levels during the dark period, consistent with the observed starch degradation (Figure 9). In L/L at 20°C the oscillations remained, showing circadian regulation of maltose accumulation. In L/D at 4°C, the amplitude of the cycles was dramatically increased, with much higher levels during the dark period, while the lowest levels were comparable at 20°C and 4°C. Interestingly, in L/L at 4°C, the initial increase of maltose was similar to the increase observed L/D at 4°C. However, these cyclic patterns were only preserved for one day, after which cycle amplitude decreased strongly. Several genes coding for enzymes involved in starch catabolism were diurnally and circadian regulated at 20°C (Figure 9). For some of these genes, such as those encoding α-amylase, the debranching enzyme *ISA3* and a cytosolic transglucosidase *DPE2*, higher expression was observed towards the end of the light period, preceding starch degradation, suggesting transcriptional regulation. For other transcripts the highest accumulation occurred during the dark period, which is not consistent with transcriptional regulation. During cold the expression of several genes of starch catabolism increased and interestingly, for those genes with expression patterns consistent with transcriptional regulation, subtle oscillations were conserved, while others showed no oscillations. However, none of the transcriptional changes were fast enough to explain the rapid accumulation of maltose at the initial time points in the cold.

### Disruption of the circadian clock impairs cold acclimation

**Figure 10:**
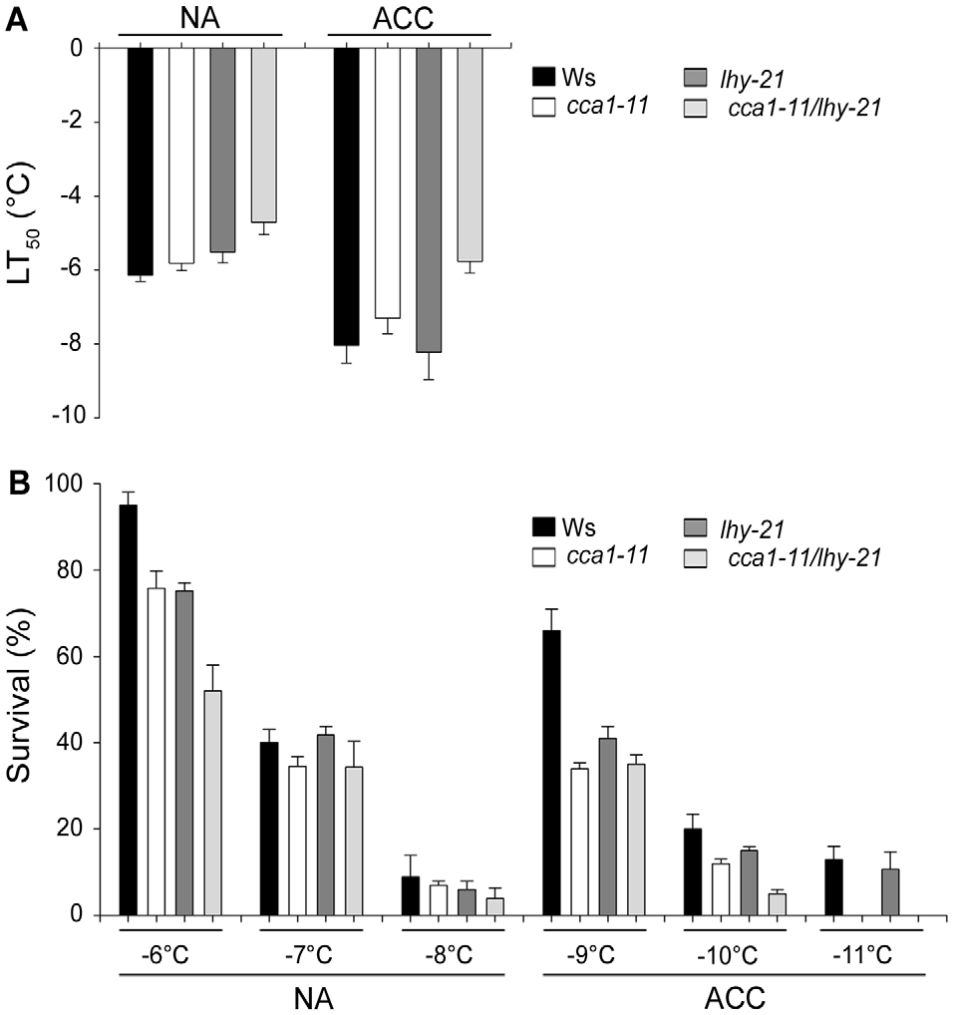
Freezing tolerance of circadian mutants *cca1-11*, *lhy-21* and *cca1-11/lhy-21* compared to the wild-type Ws. A, Electrolyte leakage was measured in whole seedlings frozen at different temperatures, either before (NA) or after cold acclimation (ACC). A total of four replicates were measured. B, The survival of the circadian mutants after exposure to freezing temperatures was estimated as the percentage of NA and ACC seedlings surviving the different temperatures after 7 d of recovery under control conditions.

To test the relevance of circadian regulation for cold acclimation we used mutants in the partially redundant MYB transcription factors LHY and CCA1 that are central components of the circadian oscillator. The *cca1-11*, *lhy-21* and *cca1-11/lhy-21* mutants were compared against their wild-type control (Ws) both using an electrolyte leakage assay and a seedling survival assay (Figure 10). In the electrolyte leakage assay the freezing tolerance of *lhy-21* and *cca1-11* was slightly lower than that of wild-type plants under non-acclimated conditions, whilst the *cca1-11/lhy-21* double mutant was clearly less tolerant, with a LT_50_ 1.4°C higher than wild-type. After cold acclimation, *lhy-21* and Ws had similar freezing tolerance whilst *cca1-11* was 0.7°C less tolerant and the double mutant was markedly less tolerant still, with a LT_50_ 2.3°C higher than wild-type. Together these data also show that the capacity of the *cca1-11/lhy-21* mutant to cold acclimate was 0.9°C less than wild-type plants. Similar results were obtained from the seedling survival assays, where *cca1-11* and *cca1-11/lhy-21* showed less survival, especially after cold acclimation.

## Discussion

### Contribution of diurnal and/or circadian regulation to the global changes during cold acclimation

Cold acclimation is a complex process that allows plants to increase their freezing tolerance and to adapt their metabolism to growth and development at low temperatures. It should be stressed in this context that for a chilling tolerant plant such as Arabidopsis, exposure to 4°C is not lethal, as plants continue to grow and eventually flower and set seed. Furthermore, significant cold acclimation occurs within 24 h under our experimental conditions [1], thus demonstrating the relevance of interpreting the observed molecular changes in the context of cold acclimation. One striking effect of low temperature is the disruption of circadian clock function, indicated by dampened or disrupted cycles of many clock components and output genes, not only in Arabidopsis [1], but also in a tree [23], [24]. This indicates that the findings presented here for Arabidopsis will be relevant for a large number of both annual and perennial plant species.

Diurnal regulation of primary metabolism, such as starch [30], [32] and sugar metabolism in leaves [29], [33] and nitrogen metabolism [34], has been previously demonstrated, mainly under short-day conditions. Under long-day conditions, we confirmed the diurnal regulation of sugar and amino acid metabolism and extended the list of molecules known to show diurnal cycles in abundance. In contrast, only few studies have explored the circadian regulation of metabolism, for example, addressing a relationship between circadian clock and TCA cycle [26], or mainly focusing on starch synthesis and break-down [29], [35]. Here, we present a comprehensive analysis of circadian regulation of central metabolism, indicating that about 30% of all analyzed metabolites showed circadian oscillations in their pool sizes. Comparison of the metabolic networks derived from the diurnal and circadian time courses indicated more connected and complex regulatory patterns under diurnal conditions. It is tempting to speculate that the higher growth rates observed for plants growing under a harmonized diurnal cycle [16] may be related to this more coordinated functioning of the complex cellular metabolic networks and that this may represent a major selective advantage of circadian function in all organisms.

The circadian clock in Arabidopsis can compensate for changes in environmental temperature over a fairly wide range (approximately 12°C to 27°C) [36]. Exposure to 4°C apparently exceeds the limits of this temperature compensation, as demonstrated previously [1]. In addition, the present data show that cold had a massive influence on the metabolome, both under circadian and diurnal conditions. However, when metabolite levels increased in response to cold, diurnal oscillations observed in nonacclimated leaves were largely preserved, while the circadian regulation of metabolite levels was interrupted (with the partial exception of maltose), due to the disruption of circadian clock function.

### Regulation of compatible solute metabolism

Many organisms accumulate compatible solutes such as different sugars and some amino acids under various stress conditions. In plants, these include proline, galactinol, raffinose and GABA [12]. They may act either nonspecifically as osmolytes, or as stabilizers for proteins and membranes, e.g. during freezing and drying [37], [38], [39], [40], [41], [42], [43], [44], [45] and GABA has additionally been discussed in relation to a role in stress signaling [46], [47]. Maltose, as a starch degradation product, has usually not been considered as a compatible solute, although it is able to stabilize membranes during desiccation [48] and was shown, either alone or by increasing other soluble sugars, to contribute to protecting photosynthetic electron transport during freezing stress [49]. The genes encoding enzymes from the biosynthetic pathways of some of these metabolites have been established as cold-regulated [50], [51]. More recently, parallel transcript and metabolite profiling indicated that the regulation of galactinol, raffinose and GABA, but not proline, were consistent with transcriptional regulation [12]. Our data confirmed the role of transcriptional regulation for these compatible solutes. Based on our metabolic profiling, it was possible to determine that accumulation of compatible solutes, such as GABA and proline (Figure 6) occur later in comparison with other compatible solutes, for example, raffinose, which itself closely follows the increase in galactinol (Figure S2). Accumulation of raffinose is in agreement with the earlier increase in the transcript encoding for galactinol synthase GolS3, which has been shown to be important in response to cold [51], and which our data shows is closely regulated together with the raffinose synthase SIP1 (Figure S2). Interestingly, raffinose cycles in L/D at 20°C (Table 1), consistent with the high amplitude cycles of GolS3 in this condition (Figure S2) and although low-amplitude cycles are visible in L/D at 4°C these do not result in significant changes in raffinose in the context of the high levels induced by cold. In the following, we will focus the discussion to some novel aspects of the proline and maltose accumulation pathways.

The synthesis of proline can be achieved by two different pathways, one starting from glutamate and one from ornithine. The reduction of glutamate by *P5CS* is the rate-limiting step in proline biosynthesis and *P5CS* is primarily regulated at the transcriptional level and by feedback inhibition by proline [52]. The expression of *P5CS* was diurnally and circadian regulated in nonacclimated leaves, while during cold acclimation the steady-state level of *P5CS* transcripts increased without diurnal oscillations. Transcripts of the ornithine-oxo-acid transaminase (δ-*OAT*) gene, coding for the enzyme involved in proline synthesis from ornithine was not diurnally or circadian regulated and was down-regulated by cold (Table S2). This indicates that the glutamate pathway through *P5CS* is mainly responsible for proline accumulation induced by low temperature, as was observed during osmotic stress [52].

### Insight into the key role of starch for growth and for cold acclimation

Maltose is a product of starch metabolism that has been shown to be under diurnal and circadian regulation [28], [29], [35]. Breakdown of starch provides a carbon source for the dark period and its rate is highly controlled to assure sustained carbon supply to metabolism during the night [30]. Our data show that in L/D 20°C, starch breakdown proceeds from dusk and is followed by peak maltose accumulation in the middle of the night which subsequently declines. Under continuous light, we did not detect clear circadian regulation of starch levels, but maltose was circadian regulated with delayed phase – peaking around 8 h later than in L/D 20°C. As the highest growth rate occurs during late night/dawn in L/D cycles, whilst growth occurs during the day and peaks at dusk in continuous light [53], [54], our data are consistent with the hypothesis of starch breakdown fuelling growth. Furthermore, based on our correlation network analysis it was evident that maltose was either negatively or positively correlated with amino acid pool sizes under diurnal or circadian conditions, respectively. This suggests that in L/D conditions demand for protein synthesis for growth is coupled to carbon supply from starch breakdown, leading to a strong decline in amino acid content during the night. However, in continuous light, the growth phase is shifted to the day and amino acid pools are no longer depleted. A decreased demand for amino acids during subjective night may therefore be responsible for the reduced amplitude of their rhythms in circadian conditions.

In common with growth, the process of cold acclimation requires considerable resources. In this respect it is interesting that cold acclimation is preceded by maltose accumulation [49] and we additionally show that maltose shows strong night-specific accumulation during cold acclimation in light-dark cycles. It is also noteworthy that maltose was the only metabolite that showed any detectable cycles in L/L at 4°C, persisting for ∼24 h. The maintenance of circadian regulation even for just 24 h may be sufficient to be of biological relevance for cold acclimation under natural light-dark conditions. Furthermore, in L/D conditions at 4°C starch degradation still occurred during the night and maltose showed similar phase of accumulation. These data all point to a key role for starch breakdown during the process of cold acclimation. This finding is well validated by the demonstration that two mutants that are impaired in starch breakdown have reduced ability to cold acclimate [49], [55].

In the context of these findings, it is interesting to consider how starch breakdown is regulated. During the initial steps of starch catabolism in chloroplasts, the polymers are degraded by α-amylases. Then, debranching enzymes (isoamylases) generate linear glucans, which are soluble in the stroma and can be further metabolized to maltose by β-amylases. In Arabidopsis, three genes encode α-amylase enzymes, but it is unclear which are necessary for starch degradation. During cold acclimation, the diurnal oscillations of one of these genes were dramatically reduced in amplitude. A unique gene encoding an isoamylase has been associated with starch catabolism (*ISA3*) and diurnal oscillations of the corresponding transcripts were also stopped during cold acclimation, while transcripts accumulated to high levels. The expression of β-amylase genes peaked during the dark period, or during subjective night in L/L conditions. During cold acclimation, the expression of two of the four β-amylase genes (*BAM2*/*BMY9* and *BAM3*/*BMY8*) increased without diurnal cycles. *BAM3*/*BMY8* has been shown previously to be induced by low temperature and its contribution to maltose accumulation during cold acclimation has been demonstrated by the use of RNAi lines [49]. Due to the diurnal and circadian oscillations of these transcripts, it was suggested that a primary regulation of the pathway could be at the transcriptional level [35]. This would be consistent with the expression pattern of genes such as α-amylases and *ISA3*, with the highest expression level toward the end of the light period, preceding starch degradation, but other genes showed different expression patterns and there is no evidence for oscillations in the amount of the encoded enzymes [28]. Therefore, other regulatory mechanisms have been invoked [32]. Our data from the L/D 4°C series clearly show that transcriptional regulation of starch breakdown is unlikely to be important in the short-term during cold acclimation.

The protection of the photosynthetic electron transport chain *in vitro* by maltose [56] and the reduced PSII efficiency in lines with *BAM3*/*BMY8* expression reduced by RNAi [49] are consistent with a potential role for maltose as a compatible solute functioning in the chloroplast. There are several studies of the subcellular localization of maltose in different plant species [57], [58], [59], [60], [61], showing it can be found in different subcellular compartments including the choloroplast, cytosol and vacuole. Thus, it is possible that maltose could act as a compatible solute in multiple compartments and have further roles beyond the protection of photosynthesis, for example in protecting other cellular membranes. However, as the distribution of maltose between cellular compartments can vary in response to light conditions [57], [58], [59], [60], [61], and we show that the strongest accumulation occurs coincident with starch breakdown in the dark (Figure 9), such dramatic changes in abundance and localization are not easily reconciled with a role as a compatible solute. Thus, it is likely that the main driver of maltose accumulation during cold acclimation is related to its intermediary role in metabolism.

### Amino acid biosynthesis pathways

The aspartate-derived amino acid pathway is a central regulator of plant growth [62]. At the transcriptional level, *ASN1* was the only gene with altered expression during cold acclimation. No other significant changes were detected that could be related to channelling of aspartic acid into methionine. Thus, post-transcriptional regulation must be responsible for the increase in methionine pool size in the cold. The first committed step in this pathway is catalyzed by a bifunctional aspartate-kinase-homoserine-dehydrogenase (AKII-HSDHII), whose activity is under positive allosteric control by alanine, cysteine, isoleucine, serine and valine [31]. During cold acclimation, increased AKII-HSDHII activity could be caused by higher levels of alanine and valine. However, their levels rose within 2-6 h, while homoserine levels only increased after 20 h - an unusually long delay for allosteric regulation. In addition, the accumulation of both homoserine and methionine during cold acclimation is in good agreement with previous studies showing that the availability of homoserine is a requirement for methionine synthesis [63]. Moreover, it has been shown that the activity of one isoform of aspartate kinase (AK2) is negatively regulated by lysine [31]. The decrease in lysine levels during cold acclimation is consistent with a release of this inhibition. Diurnal cycles were observed for homoserine and methionine, but not for other amino acids of this pathway and only these two amino acids were also accumulated during cold acclimation. Diurnal oscillations were lost when the maximal accumulation was reached. Thus, diurnal control may also participate in the regulation of the pathway, but the extent of this contribution remains unclear.

The synthesis of the branched-chain amino acids valine and leucine and the closely related isoleucine biosynthesis pathway share four common enzymes. Consistent with this, in non-acclimated leaves, the network analysis indicates these three amino acids are tightly correlated (Figure S1). Furthermore, our data show that valine, leucine and isoleucine accumulation are circadian regulated in L/L 20°C and likely show diurnal cycles in L/D 20°C (Figure S3). Transcripts of genes coding for the common steps in valine and leucine biosynthesis, as well as genes coding for isoleucine biosynthesis are not diurnally or circadian regulated (Table S1). However, two genes encoding enzymes in the degradation pathway of the valine/leucine intermediate 2-keto-isovalerate show diurnal cycles in L/D 20°C (Figure S3) which may contribute to the observed regulation.

In the cold, the network analysis revealed the three amino acids remain correlated in L/L at 4°C (Figure S1) but are no longer circadian regulated, further indicating cold disrupts circadian regulation of metabolism. Interestingly, although correlated over time, they show different cold-responses, with valine increasing in L/L at 4°C up to 2-fold in comparison to L/L at 20°C, whilst leucine shows a more modest increase and isoleucine remains largely unchanged. Significantly, despite the clear correlation of this pathway in other conditions, valine becomes uncorrelated with leucine and isoleucine in L/D at 4°C (Figure S1) indicating it is possible to uncouple their tight co-regulation and that this occurs during normal cold acclimation. Cold acclimation under L/D at 4°C, similarly to L/L at 4°C, caused valine levels to increase without corresponding changes in leucine or isoleucine, again pointing to a unique effect of cold on valine metabolism. Furthermore, the phase of valine cycles was shifted to the dark period in L/D at 4°C relative to L/D at 20°C, whilst leucine and isoleucine were more similar to L/D at 20°C. The transcripts encoding the two degradation enzymes discussed earlier both decrease and become arrhythmic in in L/D at 4°C but the speed of their regulation is probably not fast enough to explain the increase in valine. Also despite their repression by cold treatment, a loss-of-function mutant in one of these enzymes (CHY1) was recently shown to be more sensitive to freezing following cold acclimation [64].

OAS, a direct precursor of cysteine, is synthesized from serine by the activity of serine acetyltransferase (*SAT*), which is converted to cysteine by *O*-acetylserine (thiol) lyase (*OASTL*). It has been proposed that OAS acts as part of the regulatory network signaling metabolic demand for sulfur-containing compounds [65]. Glutathione is the most abundant thiol compound in plant cells, playing a major role as an anti-oxidant during stress [65]. It is therefore possible that the increase in OAS levels during cold acclimation is related to increased freezing tolerance through the anti-oxidant defense system. Consistent with this, one gene encoding glutathione reductase (*At3g24170*) and three genes encoding glutathione peroxidases (*At4g11600*; *At4g31870* and *At2g30860*) were cold induced (Table S2). Several regulatory mechanisms have been described for the cysteine biosynthesis pathway, involving both transcriptional and post-transcriptional mechanisms [66] and the overexpression of *SAT* in transgenic plants correlates with increased levels of OAS [66]. In plants at 20°C, we found coordinated diurnal and circadian oscillations in the expression of *SAT1* that were dampened and shifted during cold acclimation. In contrast, *SAT3* showed circadian oscillations uncoordinated with its more complex diurnal changes under control conditions, but diurnal regulation was more apparent when SAT3 expression was up-regulated in the cold. OAS levels showed diurnal but not circadian oscillations, which were maintained while OAS accumulated during cold acclimation. This could indicate that diurnal cycling of *SAT1* and *SAT3* may contribute to the diurnal cycles in OAS levels under both control and cold conditions.

### Implications for plant cold acclimation

Our data emphasizes the importance of having a diurnal perspective when plant stress responses are characterized. Furthermore, they provide a detailed overview of which of the previously catalogued cold-responsive metabolites and transcripts are truly cold-responsive and which are changed indirectly due to the affect of cold on the circadian clock or via diurnal regulation. The diurnal and circadian cycles observed for both metabolite and transcript levels at low temperature and their possible significance for cold acclimation are intriguing. It could involve effects on growth at low temperature, as it has been shown for the contribution of the circadian clock to plant growth under control conditions [16]. This, however, has not been explicitly investigated so far. In addition, we and others have demonstrated that circadian clock function may have direct effects on the ability of plants to increase their freezing tolerance during cold acclimation. A triple mutant in the clock component genes *PRR9*, *PRR7* and *PRR5* has increased freezing tolerance and accumulates more of the compatible solutes proline and raffinose [26], [27]. On the other hand, the *gi-3* mutant has been reported to have reduced constitutive and acclimated freezing tolerance [67]. Here, we demonstrate that the *cca1-11* and particularly the *cca1-11/lhy-21* mutants were more sensitive to freezing and were less able to cold acclimate in comparison to wild-type plants (Figure 10). Interestingly, this is in agreement with recent data from *Populus*, demonstrating that RNAi down-regulation of *LHY* orthologs reduced the ability to cold acclimate [68] indicating a potentially key role in winter dormancy responses. These data indicate that the interactions observed here between circadian and cold regulation at the molecular level are likely a relevant component of cold acclimation. The reasons for the contrasting phenotypes of different clock mutants are not presently understood, although they may relate to differences in the transcriptional regulation that has been proposed to integrate the cold- and circadian-regulated pathways [69]. However, it should also be noted that the use of existing clock mutants with significant growth and developmental phenotypes and different genetic backgrounds also complicates the assessment of freezing tolerance and comparison between different mutants. Further experiments with previous [70] and new inducible clock mutants will be needed to resolve these questions and to clarify the functional role of diurnal oscillations in metabolite and transcript levels in low temperature growth and freezing tolerance.

## Materials and Methods

### Plant material and growth conditions

The protocols used were as described previously [1], [6], [71]. Briefly, *Arabidopsis thaliana* accession Columbia (Col-0) was initially grown on soil, composed of standard compost Stendererde (Stender, http://www.stender.de) and osmocote start 1 g/L (Scotts, http://www.scottsprofessional.com) for four weeks in short days (8 h light/16 h dark) before transfer to long days (16 h light/8 h dark) at a day/night air temperature of 20°C/18°C and 150 µmol m^−2^ s^−1^ light. Experiments were started when the rosette was mature (40–45 d after germination) and completed before the inflorescence reached 1 cm. For time course at 20°C, plants were maintained in the same light and dark conditions (L/D 20°C) or transferred to 150 µmol m^−2^ s^−1^ continuous light (L/L 20°C) (Figure 1). Cold treatment was at an air temperature of 4°C and a light intensity of 90 µmol m^−2^ s^−1^, but photoperiod was either 16 h (L/D 4°C) or continuous light (L/L 4°C). Treatments were started 14 h after dawn (ZT14). Complete rosettes were harvested from five individual replicate plants, immediately frozen in liquid nitrogen and later powdered using either a ball mill (Retsch, http://www.retsch.de) or a cryogenic grinding robot (Labman Automation, http://www.labman.co.uk) [72].

### Freezing tolerance of circadian mutants

The circadian clock mutants *cca1-11*, *lhy-21* and *cca1-11/lhy-21* [36], [73], [74], [75] have early flowering phenotypes and therefore we determined freezing tolerance of seedlings prior to reproductive growth using two methods. Firstly, freezing damage of the mutants and the corresponding wild-type (Ws) was determined by an electrolyte leakage assay using the same protocol as previously described [4], [71], except that whole rosettes from individual 21 d old seedlings were used instead of detached leaves and cold acclimation was for 2 d rather than 14 d.

Secondly, seedling survival was determined as the capacity of the plants to fully recover after a freezing treatment. For this, seeds of circadian mutants and wild-type were sown in soil and grown for 2 weeks at a day/night air temperature of 20°C/18°C and 150 µmol m^−2^ s^−1^ light. Cold acclimation was performed at 4°C for 2 d as described above. Plants were first treated for 12 h at −1°C and were then exposed for 4 h to either −6°C, −7°C or −8°C (nonacclimated plants), or to −9°C, −10°C or −11°C (acclimated plants). All plants were thawed at 4°C for 24 h and then transferred to the initial growth conditions where survival was scored after 7 d.

### Transcript profiling

The five biological replicates at each timepoint were pooled and expression profiling was done as described [1], [6], [71]. Labeled aRNA was hybridized to Affymetrix ATH1 Genome arrays (ATH1) at ATLAS Biolabs (Berlin, Germany). Internal spike-in controls were included [76] and used to assess quality. There was no evidence of significant diurnal or cold-induced changes in total mRNA abundance. Data were analyzed using bioconductor [77]. Data quality was assessed with the affy [78] and AffyPLM packages. The GCRMA algorithm was used to obtain expression estimates [79]. Only transcripts that were assigned to a present call at two sequential time points in at least one time series were retained for further analysis. However, as transcripts were only required to be present in at least one time series, some included transcripts may be below the detection threshold in the other time series. Due to the process of GCRMA (quantile) normalization these transcripts are assigned a low “constant’ number, but these are unlikely to be identified as significantly regulated by our analyses. Microarray data from this article have been deposited in the ArrayExpress database (http://www.ebi.ac.uk/arrayexpress/) under accession number E-MEXP-2526.

### Metabolic profiling

Polar metabolites were extracted and analyzed by GC-TOF-MS as described [80] using an Agilent 7683 series autosampler (Agilent Technologies, http://www.agilent.com), coupled to an Agilent 6890 gas chromatograph - Leco Pegasus 2 time-of-flight mass spectrometer (LECO, http://www.leco.com). Chromatogram acquisition parameters used were as described [81]. Chromatograms were exported from Leco ChromaTOF software (version 3.25) to R software. Metabolite data were analyzed using the TargetSearch package [82]. Data were normalized by dividing each metabolite value by the median of all values for this metabolite measured in the same batch followed by a normalization to the sample median and a log2 transformation [80]. Starch measurement was done as described [83]. Glucose, fructose, sucrose and raffinose were additionally quantified by HPAEC using a CarboPac PA-100 column on a ICS3000 chromatography system (Dionex) coupled with pulsed amperometric detection by a gold electrode as described [84]. In all cases, five biological replicates for each time point were included. Metabolic profiling data are available via our website (http://www-en.mpimp-golm.mpg.de/03-research/researchGroups/05-centralInfrastrGrp/Transcript_Profiling/smp/ps/index.html).

### Data analysis

PCA was performed using the pcaMethods bioconductor package [85]. The heatmap (Figure 4) was generated in Microsoft Excel using a macro kindly provided by Yves Gibon (INRA Bordeaux, France). HAYSTACK algorithm [86] was used to identify circadian regulation of transcripts and metabolites based on either 16/8 or 8/16 hours (peak/trough) models in order to match the long-day light/dark cycles used in our study (Table S2 and Figure S4). Circadian oscillations were identified using default parameters (see http://haystack.cgrb.oregonstate.edu for details) except that a fold-change (FC) of 1.5 was applied for transcripts, whilst for metabolites a correlation cutoff of 0.7 and FC of 1.2 were used. For comparison purposes, cosine 12/12 models, as generally used in circadian studies, were also applied to identify transcripts or metabolites with a symmetrical waveform (Table S2 and Figure S4). Essentially very similar molecules were selected with both models, but for the purpose of clarity we present the 12/12 results separately as supplementary data (Tables S1 and S3). Because of the greater diversity of cycle patterns, we used an autocorrelation approach for the identification of diurnal cycles. Correlations between sequential days were calculated based on four sets of six timepoints starting at 2, 6, 10 and 14 h in our timeseries (corresponding to ZT16, ZT20, ZT0 and ZT4, respectively). We considered those where the average correlation was above 0.7 as diurnally regulated if they additionally had a FC during the cycle greater than 1.5 for transcripts or 1.2 for metabolites. As each timepoint only had a single replicate in transcript profiles, cold-regulated genes were identified using limma with a sliding window of three sequential timepoints as replicates. A total of 5251 genes were selected as significantly cold responsive in at least one of the time windows (FDR<0.05). K-means clustering with Euclidean distance was performed with the software tool TIGR MEV [87] using log2 normalized data. Transcriptional changes in diurnal and circadian cycles and transcripts classification are summarized in Table S2.

For correlation network analysis, Spearman correlation coefficients were calculated in R based on log10 transformed metabolite concentrations for 16 time points and all 5 replicate plants for each of the 4 conditions. Undirected networks were constructed based on Spearman metabolite-metabolite correlation r-values with nodes representing the measured metabolites. Undirected edges were drawn between metabolites in case the Bonferroni corrected p-value of the Spearman correlation for two metabolites was <0.001 which corresponds to a correlation coefficient R of about 0.54 for the given sample size. Networks for visualization in Cytoscape [88] were constructed with the R package GeneNet [89]. Topological parameters of the networks were calculated with the Cytoscape plugin Network Analyzer [90].

## Supporting Information

Figure S1 Metabolic networks constructed for diurnal and circadian time courses. Networks were constructed based on Spearman metabolite-metabolite correlation r-values with nodes representing the measured metabolites. Undirected edges were drawn between metabolites in case the Bonferroni corrected p-value of the Spearman correlation for two metabolites was <0.001 which corresponds to a correlation coefficient R of about 0.54 for the given sample size. Blue or red edges indicate significant positive and negative pairwise correlations, respectively.
(9.37 MB TIF)

Figure S2 Coordinated transcriptional regulation of raffinose and putrescine. Summary of the metabolic pathway, transcript and metabolite profiles of raffinose (a) and putrescine (b). For transcripts, relative expression (log2) from a pool of five biological replicates is indicated. Metabolite accumulation (log2) corresponds to the normalized peak apex intensities from five biological replicates. GOLS3, galactinol synthase 3 (At1g09350); SIP1, raffinose synthase (At5g40390); ADC, arginine decarboxylase 1 (At2g16500) or 2 (At4g34710); At-AIH, agmatine iminohydrolase; N-carbamolyputrescine amidohydrolase.
(3.63 MB TIF)

Figure S3 Integration of gene expression and metabolite accumulation for the branched-chain amino acids valine, leucine and isoleucine. Summary of metabolic pathways, transcript and metabolite profiles of the branched-chain amino acids valine, leucine and isoleucine. For transcripts, relative expression (log2) from a pool of five biological replicates is indicated. Metabolite content (log2) corresponds to the normalized peak apex intensities from five biological replicates. Atbcat, branched-chain amino acid aminotransferase; branched-chain keto-acid dehydrogenase complex includes LPD2 (At3g17240), BCE2 (At3g06850), At5g09300 and At3g13450 genes; CHY1, 3-hydroxyisobutyryl-CoA hydrolase 1 (At5g65940).
(3.58 MB TIF)

Figure S4 Models used to identify circadian patterns in transcripts and metabolites. Two asymmetric models (asymm_16/8 and asymm_8/16) that correlate with the experimental conditions (16 h light/8 h dark cycles) were used for the HAYSTACK analysis to identify circadian regulated transcripts and metabolites (see methods). For comparison purposes, cosine models, as generally used in circadian studies (with 12 h light/12 h dark cycles), were also applied to identify transcripts or metabolites with a symmetrical waveform. For the identification of circadian patterns in transcripts and metabolites, models were shifted in 1 hour, generating a change in phase that covered the complete 24 hour period. For simplicity, only models with phase equal to zero are shown in the figure. Also note, as like other circadian studies, our datasets are sampled with 4 h resolution and thus the models in Table S3 only represent a subset of the timepoints shown in this figure.
(0.83 MB TIF)

Table S1 Summary of transcriptional changes showing diurnal and circadian cycling genes, as well as cold responsive transcripts. (A) Only transcripts that were assigned a present call at the same time of day (for diurnal and circadian control datasets) or 2 subsequent timepoints (for cold treated plants) in at least 1 of the 3 time series are included. (B) Sequence similarity to the genes in the non-redundant protein database of GenBank and the database of translated protein sequences from TAIR 6.0 release. (C) Diurnal time series at low temperature: Light and dark cycles (16 h/8 h) at 4°C. (D) Diurnal time series in control temperature: Light and dark cycles (16 h/8 h) at 20°C. (E) Circadian time series in control temperature: Continuous light at 20°C. In (C),(D) and (E) numbers correspond to normalized log2 values. (F) Identification of diurnal cycles was done with autocorrelation analysis. TRUE means the gene matches the criteria of showing diurnal oscillations. See Materials and Methods for details. (G) Identification of circadian cycles was done using the Haystack algorithm (Michael et al., 2008), TRUE means the gene matches the criteria of showing circadian oscillations. (H) Identification of significant cold responsive genes consider a FDR<0.05, TRUE means the gene matches the criteria of being cold responsive. (I) K-means clustering was used for select genes with oscillations at low temperature, TRUE means the gene matches the criteria of showing cycles in cold. For (G), (H) and (I) see Materials and Methods for details.
(7.43 MB XLS)

Table S2 Model patterns used to identify circadian oscillations in transcripts and metabolites. The Haystack algorithm (Michael et al., 2008) was used to determine the correlation of our experimental data (transcripts and metabolites from circadian time series) with a oscillatory models. Each row correspond to a different model pattern.
(0.04 MB XLS)

Table S3 Comparison between different models applied for the identification of circadian metabolites. The Haystack algorithm (Michael et al., 2008) was used to determine the correlation of our experimental metabolite data from circadian time series. Since the plants were entrained using a 16/8 cycles, models depicting 16/8 hours were used to identify significant biological rhythms. For comparsion with previous published data, a oscillatory 12/12 hours cosine model was included.
(0.04 MB XLS)
