# Supplementary figures and images for: Interaction with Diurnal and Circadian Regulation Results in Dynamic Metabolic and Transcriptional Changes during Cold Acclimation in Arabidopsis
Source: PLoS One. 2010 Nov 23;5(11):e14101. doi: 10.1371/journal.pone.0014101 (PMC2990718; doi:10.1371/journal.pone.0014101)

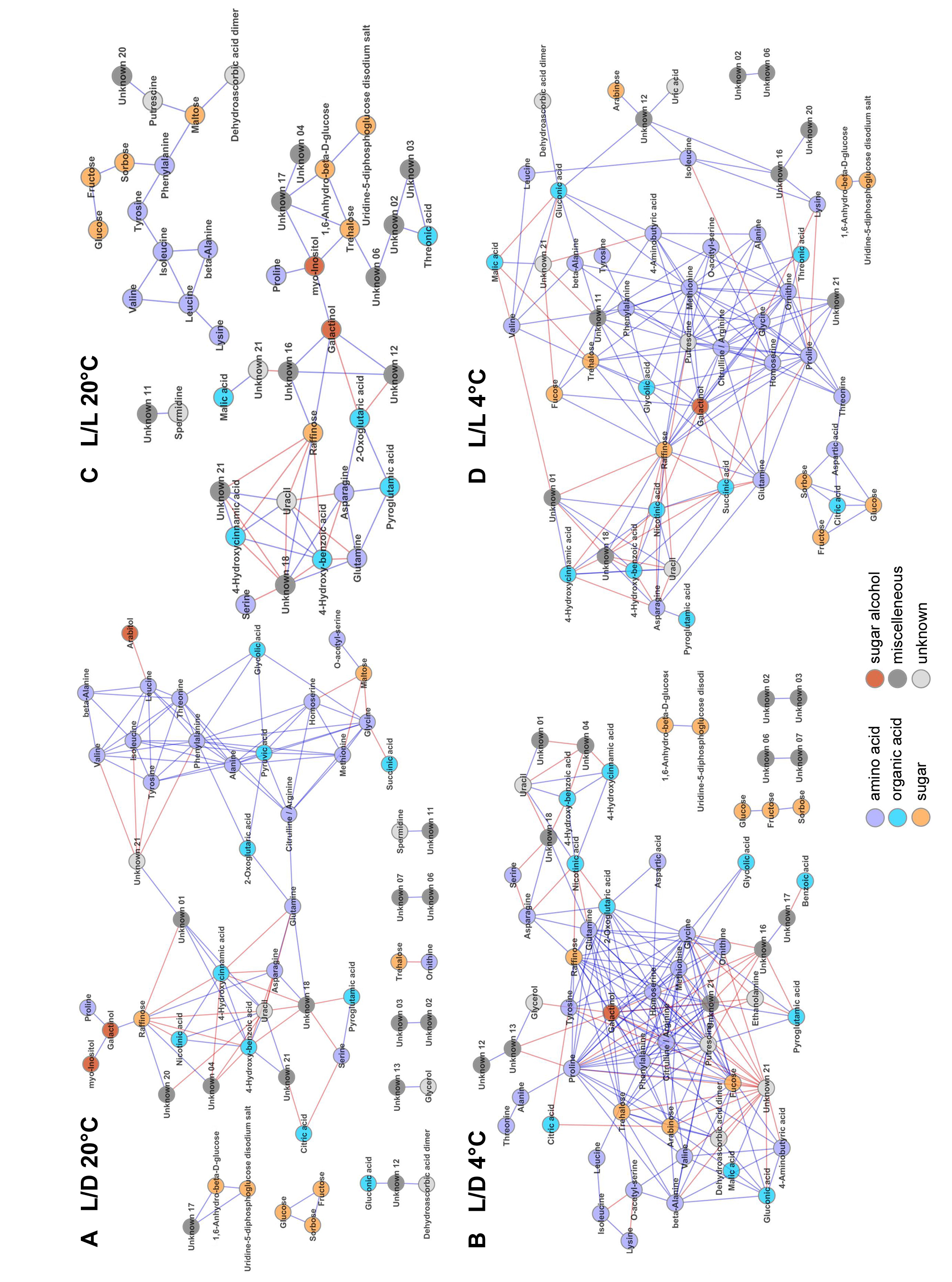

Supplement: Figure S1 — Metabolic networks constructed for diurnal and circadian time courses. Networks were constructed based on Spearman metabolite-metabolite correlation r-values with nodes representing the measured metabolites. Undirected edges were drawn between metabolites in case the Bonferroni corrected p-value of the Spearman correlation for two metabolites was <0.001 which corresponds to a correlation coefficient R of about 0.54 for the given sample size. Blue or red edges indicate significant positive and negative pairwise correlations, respectively. (9.37 MB TIF) [file pone.0014101.s001.tif]

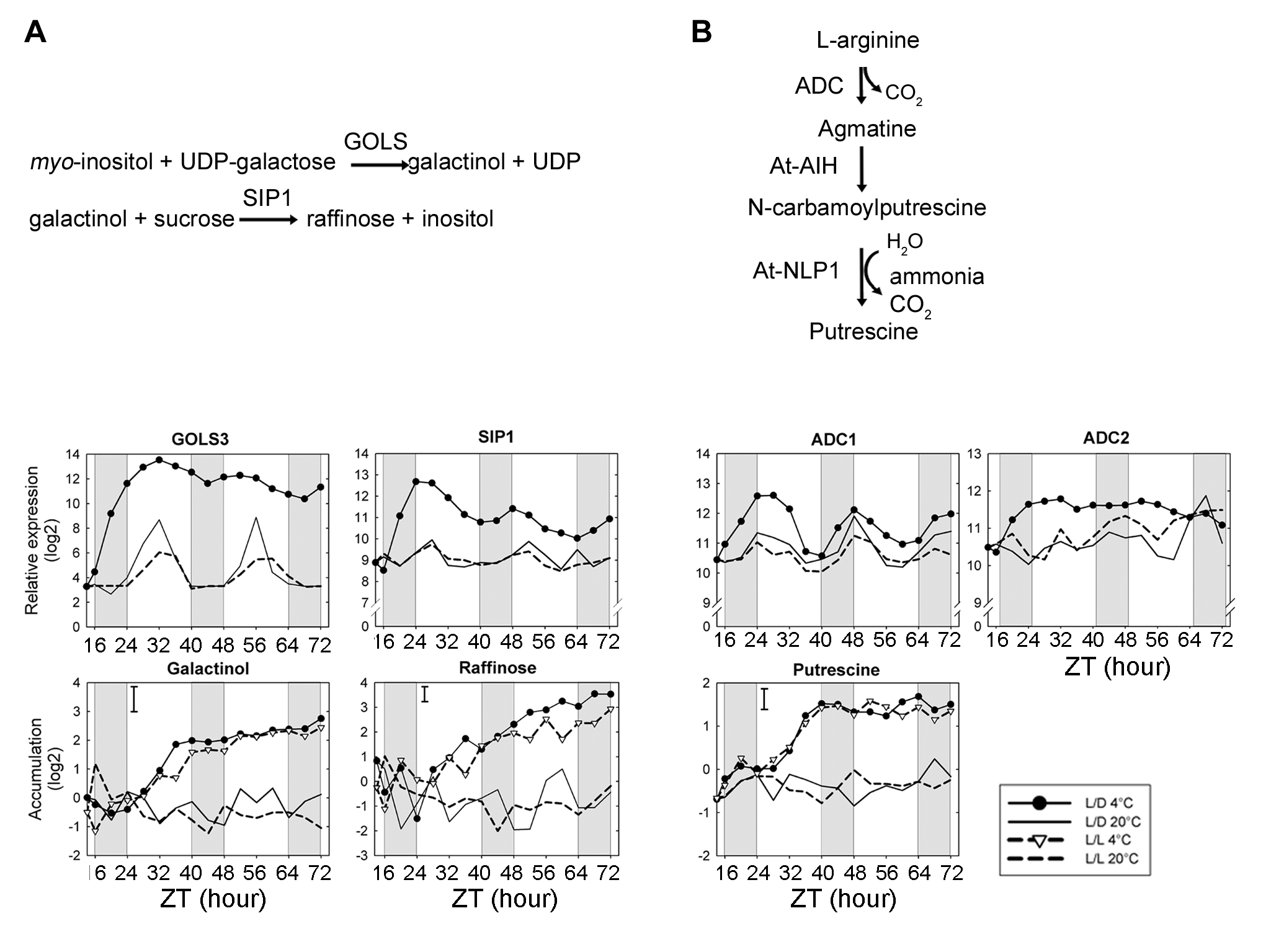

Supplement: Figure S2 — Coordinated transcriptional regulation of raffinose and putrescine. Summary of the metabolic pathway, transcript and metabolite profiles of raffinose (a) and putrescine (b). For transcripts, relative expression (log2) from a pool of five biological replicates is indicated. Metabolite accumulation (log2) corresponds to the normalized peak apex intensities from five biological replicates. GOLS3, galactinol synthase 3 (At1g09350); SIP1, raffinose synthase (At5g40390); ADC, arginine decarboxylase 1 (At2g16500) or 2 (At4g34710); At-AIH, agmatine iminohydrolase; N-carbamolyputrescine amidohydrolase. (3.63 MB TIF) [file pone.0014101.s002.tif]

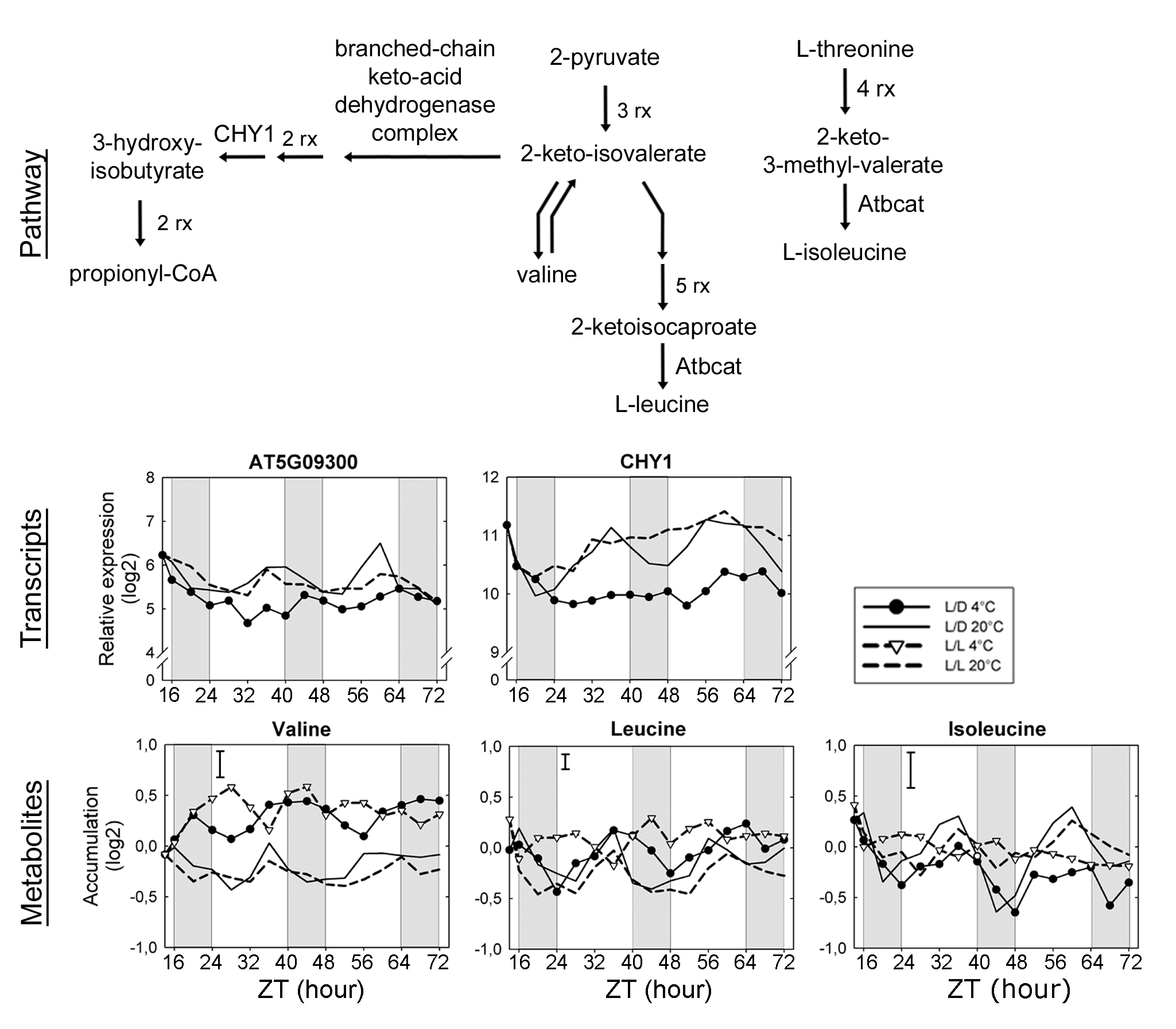

Supplement: Figure S3 — Integration of gene expression and metabolite accumulation for the branched-chain amino acids valine, leucine and isoleucine. Summary of metabolic pathways, transcript and metabolite profiles of the branched-chain amino acids valine, leucine and isoleucine. For transcripts, relative expression (log2) from a pool of five biological replicates is indicated. Metabolite content (log2) corresponds to the normalized peak apex intensities from five biological replicates. Atbcat, branched-chain amino acid aminotransferase; branched-chain keto-acid dehydrogenase complex includes LPD2 (At3g17240), BCE2 (At3g06850), At5g09300 and At3g13450 genes; CHY1, 3-hydroxyisobutyryl-CoA hydrolase 1 (At5g65940). (3.58 MB TIF) [file pone.0014101.s003.tif]

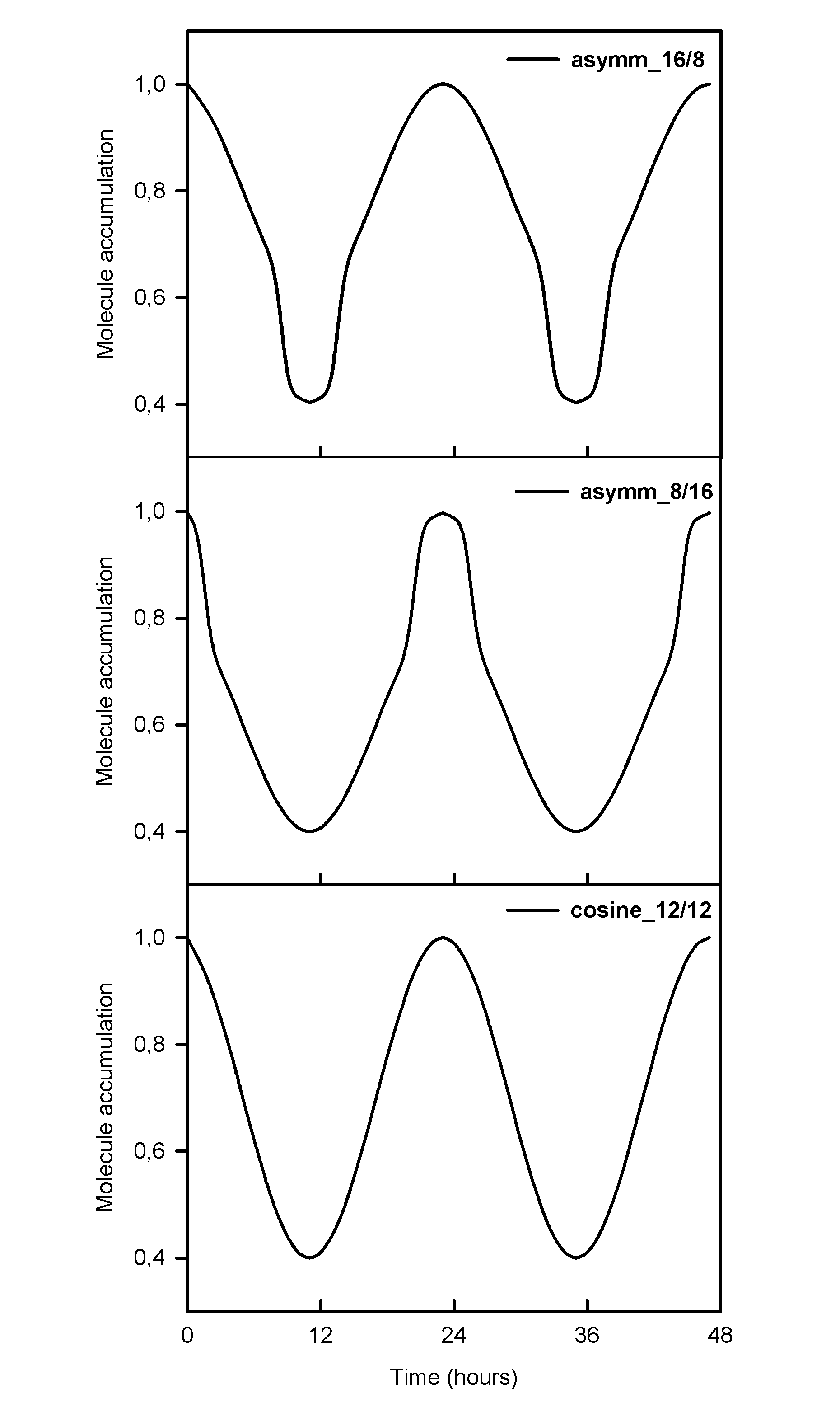

Supplement: Figure S4 — Models used to identify circadian patterns in transcripts and metabolites. Two asymmetric models (asymm_16/8 and asymm_8/16) that correlate with the experimental conditions (16 h light/8 h dark cycles) were used for the HAYSTACK analysis to identify circadian regulated transcripts and metabolites (see methods). For comparison purposes, cosine models, as generally used in circadian studies (with 12 h light/12 h dark cycles), were also applied to identify transcripts or metabolites with a symmetrical waveform. For the identification of circadian patterns in transcripts and metabolites, models were shifted in 1 hour, generating a change in phase that covered the complete 24 hour period. For simplicity, only models with phase equal to zero are shown in the figure. Also note, as like other circadian studies, our datasets are sampled with 4 h resolution and thus the models in Table S3 only represent a subset of the timepoints shown in this figure. (0.83 MB TIF) [file pone.0014101.s004.tif]
